# RESTOP: Retaining External Peripheral State in Intermittently-Powered Sensor Systems

**DOI:** 10.3390/s18010172

**Published:** 2018-01-10

**Authors:** Alberto Rodriguez Arreola, Domenico Balsamo, Geoff V. Merrett, Alex S. Weddell

**Affiliations:** Department of Electronics and Computer Science, University of Southampton, Southampton SO 17 1BJ, UK; d.balsamo@soton.ac.uk (D.B.); gvm@ecs.soton.ac.uk (G.V.M.); asw@ecs.soton.ac.uk (A.S.W.)

**Keywords:** energy harvesting, external peripheral, sensor system, transient computing

## Abstract

Energy harvesting sensor systems typically incorporate energy buffers (e.g., rechargeable batteries and supercapacitors) to accommodate fluctuations in supply. However, the presence of these elements limits the miniaturization of devices. In recent years, researchers have proposed a new paradigm, transient computing, where systems operate directly from the energy harvesting source and allow computation to span across power cycles, without adding energy buffers. Various transient computing approaches have addressed the challenge of power intermittency by retaining the processor’s state using non-volatile memory. However, no generic approach has yet been proposed to retain the state of peripherals external to the processing element. This paper proposes RESTOP, flexible middleware which retains the state of multiple external peripherals that are connected to a computing element (i.e., a microcontroller) through protocols such as SPI or I${}^{2}$C. RESTOP acts as an interface between the main application and the peripheral, which keeps a record, at run-time, of the transmitted data in order to restore peripheral configuration after a power interruption. RESTOP is practically implemented and validated using three digitally interfaced peripherals, successfully restoring their configuration after power interruptions, imposing a maximum time overhead of 15% when configuring a peripheral. However, this represents an overhead of only 0.82% during complete execution of our typical sensing application, which is substantially lower than existing approaches.

## 1. Introduction

**Figure 1:**
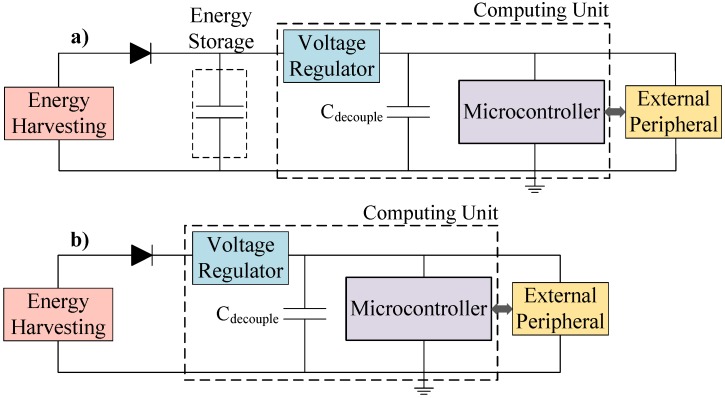
Schematic of: (**a**) an energy-neutral; and (**b**) a transient, EH sensor system.

Energy harvesting (EH) potentially enables the long-term deployment of low-power sensor systems without the need to replace batteries. However, EH sources are usually intermittent and unpredictable because they depend on external conditions (i.e., availability of energy to be harvested) [1]. To overcome this limitation, systems typically integrate energy storage devices (e.g., supercapacitors or rechargeable batteries) to smooth out supply variations. This approach is known as energy-neutral operation, where energy storage is used to balance the stored energy with the long-term energy consumed and, thus, sustain operation during power shortages [2]. This is shown in Figure 1a, where energy storage is used to sustain computation when there is insufficient energy being harvested. However, energy storage increases the system’s cost, size and mass. Transient computing (Figure 1b) aims to power systems directly from the EH source, operating when energy is available and retaining system state during supply interruptions.

Various software solutions for transient computing have coped with power intermittency by saving the system state (contents of main memory, core and general purpose registers) into a Non-Volatile Memory (NVM) [3,4,5,6,7]. Thus, after a supply interruption, the system state is restored and the program continues from the point where it was interrupted, instead of restarting from the beginning. Recent hardware approaches overcome the need to save and restore the system’s state by using non-volatile processors [8]. These approaches are only effective in retaining the state of on-chip peripherals that are controlled by the special function registers of the microcontroller unit (MCU), e.g., internal ADCs. However, the vast majority of sensing systems also include external sensors, actuators, radio transceivers, etc. A recent approach [9] attempted to save and restore the state of external peripherals; however, it only operates with peripherals that interact with the MCU through SPI. Moreover, this approach requires the user to make several adjustments depending on the type and number of peripherals connected (it is not generic) and causes a high time overhead.

In this paper, we present RESTOP (REtaining the STate Of Peripherals), a novel middleware to retain the state of digitally interfaced peripherals in transiently-powered systems. RESTOP provides generic functions to read data from the external peripherals or write to them, keeps track of and saves the transmitted configuration data, and hence retains the peripheral state. Thus, after a power failure, the peripheral state can be restored, without requiring for the user to implement the save and restore functions for each attached peripheral and indicate the order in which they have to be restored. The key contributions of this work are:

- A novel and generic approach for transient computing systems, which retains the state of multiple digitally interfaced peripherals between power outages (Section 3).
- Implementation of the approach into a middleware (available open-source to download from http://www.transient.ecs.soton.ac.uk) that works with both SPI and I${}^{2}$C protocols (Section 4).
- A thorough practical evaluation of RESTOP in order to validate the operation of the middleware and the time overhead it causes in an intermittently-powered sensor system (Section 5).

RESTOP can be integrated into any of the existing approaches to transient computing [3,4,6,7,10]. Experiments demonstrate that RESTOP is able to retain and restore peripheral state with a peripheral configuration time overhead of up to 15%. However, this represents an overhead of only 0.82% during complete execution of our typical sensing application.

## 2. Problem Statement and Motivation

**Figure 2:**
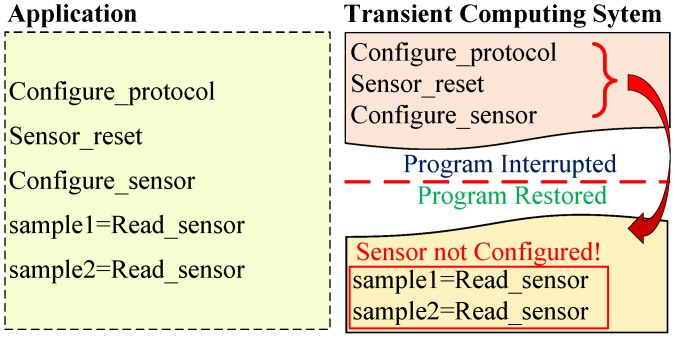
Operation of existing transient computing approaches when working with external peripherals after a power failure.

A transiently-powered sensor system could not operate properly unless the peripheral state was restored after a power outage. Figure 2 shows an example of an incorrect operation that may occur in a conventional transient system (e.g., HarvOS [10]). The application first configures the serial protocol (Configure_protocol) to communicate with the external peripheral (in this example, a digital sensor). Then, the MCU sends a reset instruction to the peripheral (Sensor_reset) and configures the sensor to start sampling data (Configure_sensor). However, before the peripheral starts sampling, a power failure occurs. Then, when the energy is again available the system state is restored. Nevertheless, the peripheral’s state is not restored, i.e., the sensor is not properly configured (the configuration was not saved into NVM). Therefore, the sensor would revert to its default configuration after the power outage, and the program would be unaware of this.

There are various software approaches to transient computing that save a copy of system state into NVM (snapshot), before a power failure [3,4,6,7,10] and restore it when the energy is again available instead of starting from the beginning. However, these solutions are focused on retaining the state of the main memory, core registers (i.e., program counter, stack pointer, link register and general purpose registers) and peripheral registers (which are used to control internal peripherals such as ADC, DAC, GPIO, etc). These approaches are not concerned with retaining the configuration of external peripherals because they are not included in the design. This has led researchers to engage in developing solutions that allow the state of external peripherals to be retained between power outages.

Berthou et al. [9] proposed Sytare, a software approach which retains not only the system state but also the configuration of external peripherals attached to the MCU through SPI. This approach includes so-called kernel code, which operates between the main application and the library to access the external peripheral features (peripheral driver), and it is in charge of saving and restoring the peripheral state. However, the user not only has to write the function to configure the peripherals but also implement a structure called device context for each attached peripheral. This structure is used by Sytare to encapsulate the functions and save the data exchanged between the MCU and the external peripheral. Moreover, the user has to write a function to restore the peripheral configuration (one per connected peripheral). If more than one peripheral is attached, the developer has to indicate in which order they have to be restored, because this solution would not work in a system where the peripherals are accessed in a different order from one cycle to another. However, it excludes essential details: it does not describe how the developer has to change each peripheral driver in order to update the structure (device context) needed to avoid peripheral volatility and how to implement the restore function for each attached peripheral. Besides that, Sytare incurs a time overhead of over 30 μs per peripheral instruction because it needs to save the peripheral state each time an instruction is issued. This imposes an overhead of up to 137% when configuring a radio transceiver.

Recently, designers have oriented their research towards implementing non-volatile solutions for external peripherals. Li et al. [11] proposed a ferroelectric non-volatile flip-flop based input-output (IO) architecture that aims to reduce the initialization overhead caused by power outages. They replaced typical IO D-type flip flops with non-volatile flip-flops by adding two ferroelectric capacitors. Thus, when a power outage occurs, the peripheral configuration is retained in local ferroelectric capacitors, allowing a fast backup operation. However, this approach is focused exclusively on sensors, requires special-purpose hardware and does not offer a solution for off-the-shelf peripherals. Hardware approaches such as Non-Volatile Processors (NVPs) [8] attempt to save *in-place* snapshots by adopting non-volatile SRAM and registers. However, these hardware approaches do not offer solutions as they only retain the system state (main memory and processor registers), but not the configuration of digitally interfaced peripherals. Other solutions such as WISP [12], WISPCam [13] or federated energy storage [14] do not snapshot the configuration of the external peripherals because they operate only when the energy stored in small capacitors is enough to complete the required task. The peripherals are configured from scratch and perform the same function each time they are enabled.

In summary, there is an unmet need for a generic solution capable of retaining the state of multiple peripherals connected to the MCU through external interfaces such as I${}^{2}$C and SPI. This would enable the state of complete sensor systems, e.g., incorporating an MCU, a digital luminosity sensor [15] and a transceiver [16].

## 3. RESTOP: A Middleware for Peripheral State Retention

**Figure 3:**
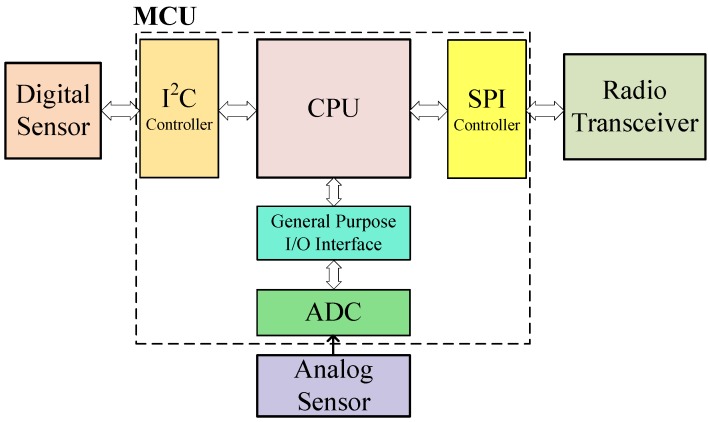
Block diagram of an MCU interacting with different peripherals.

External digital peripherals are typically connected to the MCU via serial protocols, unlike the on-chip peripherals that are controlled by special function registers. Figure 3 shows a block diagram of an MCU interacting with three peripherals: an analog sensor connected via an on-chip ADC, a transceiver connected through SPI and a digital sensor attached via I${}^{2}$C. In Section 2, the limitations of existing transient computing systems when working with digitally interfaced peripherals were described. To address them, we propose RESTOP, a middleware which is generic for different peripherals and serial communication protocols (e.g., SPI or I${}^{2}$C), capable of retaining (saving and restoring) peripheral configurations between power failures. The following terms are introduced here to aid understanding of the operating principles of RESTOP:

- Peripheral operation: The action to be performed on the peripheral, i.e., write or read.
- Peripheral instruction: The information required by the system to issue the operation on the peripheral (e.g., peripheral address, register to be read, value to be written on the register, etc.).
- Parameters: Elements that constitute each function that executes the peripheral instructions.

**Figure 4:**
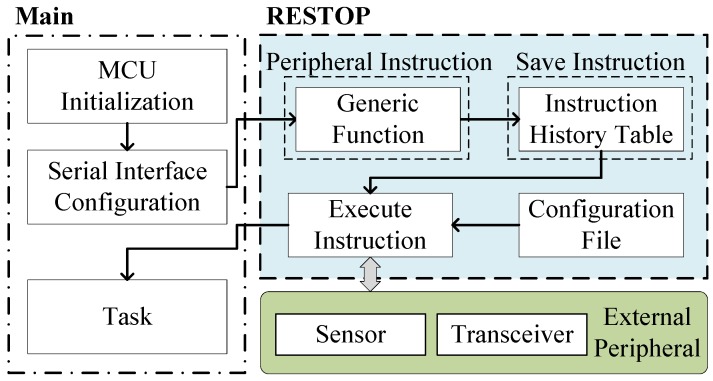
Diagram of RESTOP interacting with the application and peripherals.

Figure 4 shows the parts that make up RESTOP and how this middleware interacts in a sensor system when saving and executing a peripheral instruction (the Restore module is later described in Figure 6). Each peripheral instruction is issued through the generic functions provided by RESTOP and saved in a history table. In order to execute the instruction on the peripheral, RESTOP complements the information entered through the generic functions with that defined in a configuration file (these modules are detailed in Section 4). The history table can either be: (1) placed in main memory; or (2) directly located in NVM. In the first case, the developer can utilize any of the existing approaches for transient computing [3,6,7,10] that can save the system state (including main memory) to NVM at the right time before a power failure. Thus, after a power outage, the system state (including the instruction table) is restored and then RESTOP restores the peripheral configuration by re-issuing the instructions from the table. In the second case, RESTOP has to be included with interrupt-based approaches such as Hibernus [6] and QuickRecall [4] in order to ensure that the system and peripheral states are restored in the same point where they were interrupted, i.e., there is no more code executed after the snapshot. Thus, it is possible to maintain coherence, avoiding the table being modified after the last snapshot was saved. This is important because in a transient system with external peripherals, repeated peripheral instructions (or system code) may result in functionality issues [5]. In Section 3.1, the different factors that RESTOP considers before saving and executing a peripheral instruction are described. Later, in Section 3.2, the process of restoring the peripheral configuration is detailed.

### 3.1. Saving and Executing Peripheral Instructions

**Figure 5:**
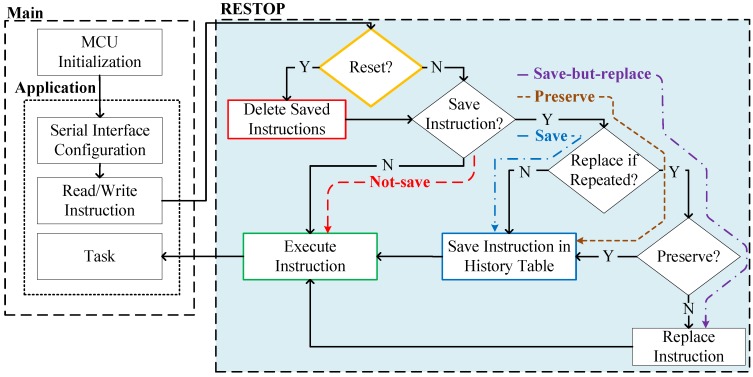
Path followed to save and execute an instruction depending on the selected criteria.

Figure 5 details the process of saving and executing a peripheral instruction issued over a serial protocol. The decision about whether RESTOP should save an instruction in the table is made by the programmer at design time for each peripheral instruction, considering four choices:

1. Not-save: The user might consider that a certain instruction should not be saved because it is not a peripheral configuration instruction (e.g., reading a status register).
2. Save: The issued instruction must be saved in the history table without checking whether a similar instruction (i.e., with same peripheral address and register value) was previously saved.
3. Save-but-replace: The issued instruction would replace any other similar instruction (i.e., same peripheral address and register value) that was previously saved in the history table.
4. Preserve: An instruction has to be kept in the history table regardless of whether a similar instruction is later issued.

As shown in Figure 5, RESTOP first checks whether the issued instruction is applying a *Reset* on the peripheral. *Reset* is a *write* peripheral instruction that, when issued, causes RESTOP to delete all instructions saved in the table for that peripheral. This condition is important for efficient memory usage because it is unnecessary to keep peripheral instructions prior to a *Reset*. Next, RESTOP checks whether the issued instruction has to be saved. If not (*Not-save*), RESTOP executes the instruction on the peripheral and the application continues to the next task. If the peripheral instruction must be saved, RESTOP considers two choices: *Save* and *Save-but-replace*. If the first option is asserted, the issued instruction is saved in the table and then executed on the peripheral. In case that *Save-but-replace* is selected, the middleware looks in the table for a similar instruction previously saved. If a similar instruction is found in the table, RESTOP checks whether the instruction is marked to be preserved (Preserve). If so, the instruction is saved in a new element in the table and then executed. Preserving an instruction, instead of replacing it, is particularly useful for certain peripherals that need an *unlock* instruction, which enables the peripheral to be accessed or configured. Thus, each unlock instruction is saved in the table no matter how many times it is repeated. If *Preserve* is not asserted, the previous instruction is deleted and the issued one is saved instead, but keeping the chronological order in which the instructions are sent. Keeping track of the instruction sequence is important because peripherals often need the registers to be accessed in a certain order to operate properly (e.g., in a transceiver, it has to set the channel before transmitting the data, not the other way around).

It is important to mention that each instruction must be saved before executing it in order to cope with power failures occurring before peripheral access is completed, avoiding consistency issues. Issuing an instruction on a serial interface involves, among other things, enabling the peripheral, sending the register to be read or written, waiting for the transmission to be completed, and disabling the peripheral. This sequence has to be completed without interruptions, i.e., if a supply failure occurs while an instruction is issued, the sequence has to be restarted from scratch when power recovers (e.g., it is not possible to send a partial packet). Therefore, if the instruction was not saved into the history table before the power failure, it would neither be properly executed because the sequence was interrupted, nor restored because it was not saved. A possible concern is that, when the peripheral is a radio transceiver, the user may send the instruction with the packet to be transmitted, but there would be no certainty that it was sent (i.e., a power outage may occur before packet transmission has completed). When restoring the transceiver state, the packet would be resent, leading to a duplicate packet being received. However, this can occur normally in a noisy wireless network, and communication protocols are typically already present to ignore duplicate packets and request those missed.

### 3.2. Restoring Peripheral State

**Figure 6:**
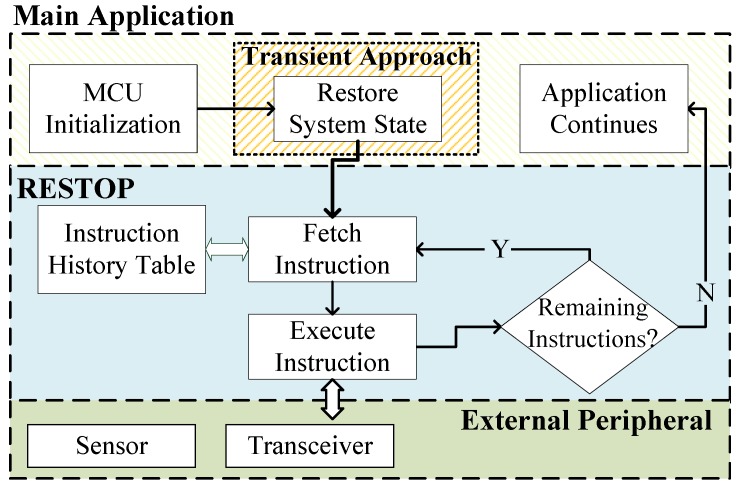
RESTOP has to re-issue each saved instruction to restore the peripheral state, after a power outage.

The restore routine is shown in Figure 6. This is executed after the system state has been restored by the transient computing approach used to protect the system from volatility. Here, RESTOP fetches each instruction from the history table and issues it over the digital interface in the correct order. This process is repeated until all saved instructions are executed, and, therefore, the state of the peripheral is restored. Once the routine is completed, the main application continues its execution from the point where it was interrupted by the power outage.

## 4. Software Algorithm Design

The requirement for a generic interface implies that it can work across different protocols and handle different types of instructions, and that it fits in not only with programming structures but also with the use cases of transient technologies. In this section, we describe the implementation of the three main elements that compose RESTOP. First, we detail the RESTOP functions that will execute the peripheral instructions (Section 4.1). Then, we list the parameters that have to be saved to properly describe each peripheral instruction without losing generality, and how the users will introduce the required information for each instruction (Section 4.2). Lastly, we explain how the instruction history table will be efficiently built in terms of time and memory usage (Section 4.3).

### 4.1. Function Implementation

Defining the functions to execute each peripheral configuration instruction, and the information to be saved from them, required the analysis of various peripherals with digital interfaces, identifying patterns that help to implement the RESTOP functions that are generic for different peripherals and serial protocols. From this analysis, we found that peripheral instructions perform two main operations: read and write. However, the number of parameters required to perform these operations varies from one peripheral to another. For example, some peripherals [17] need a 1-byte parameter called *command* to indicate the type of operation the issued instruction will perform (i.e., *read* or *write*) on a register address (i.e., the sequence would be <*command byte*><register address><data byte>). Others allow certain *single byte instructions* (no data is transferred), usually so-called *command strobes* that cause internal sequences to start in the peripheral, e.g., some peripherals have a single header byte that, when addressed, starts a self-calibration routine to define the sampling frequency [17]. Most peripherals support multiple byte transfers also known as *data burst transmissions* which send first the register address and then a sequence of different values to write to this address (this can also be applied for read operations).

Considering this analysis, we have defined the functions that will be used by the middleware to save, execute and restore each instruction:

1. RESTOP_read(): Returns the read value from the desired register.
2. RESTOP_write(): Writes a value into a peripheral register.
3. RESTOP_strobe(): Performs write operations that, unlike RESTOP_write(), executes single byte instructions.
4. RESTOP_restore(): Restores the peripheral state by executing all the instructions saved in the history table after a power failure. It has to be incorporated into the restore routine of the main application.

These functions have to be used by the developer to configure the peripherals and obtain data from them. The parameters of each function are described in Section 4.2.

### 4.2. Parameters to be Saved and Configuration File

Following the definition of the generic functions, the parameters that will constitute each peripheral instruction need to be defined. For this purpose, we separate the *dynamic* parameters that vary from one peripheral instruction to another, and those that are *static* for each peripheral attached to the system. Table 1 shows the *dynamic* parameters that will be saved in the history table. The size (number of bits) of each parameter varies depending on the information that they contain. The first parameter (*Protocol*) is a 1-bit flag to indicate the serial protocol type of each peripheral (0→SPI; 1→I${}^{2}$C). Parameter *Burst* is also a 1-bit flag that has to be set to 1 when the function will execute a *burst read*/*write* instruction. *Read* is a flag used by RESTOP to distinguish when the instruction is for a *read* (*R*=1) or *write* (*R*=0) operation. *Prv* is a 3-bit flag that can have five different values as shown in Table 2. These values are defined following the criteria described in Section 3.1. Thus, the first three options indicate whether the instruction will not be saved in the table (*Not-save*), will be saved in a new element (*Save*) or will replace a similar one if it was previously saved in the table (*Save-but-replace*). The last two criteria, shown in Table 2, indicate the instruction will be not only saved but also preserved in the table regardless of whether a similar instruction is later issued (*Preserve*).

The parameter *ID* is used to indicate the peripheral to which the saved instruction corresponds. A system may have more than one peripheral attached to the MCU, hence, we would have to identify which instruction corresponds to each peripheral. The parameters *Register* and *Value* are each one byte, corresponding to the register width of typical digital interface peripherals. *Next* and *Prev* are used to keep track of the order in which the instructions are issued. Thus, when a new instruction replaces another previously saved or is added in a new element in the table, RESTOP can keep the chronological sequence in order to properly restore the peripheral state after a power outage. The size of these parameters is one byte each too, allowing the system to map up to 256 peripheral instructions. This is considered sufficient for most peripherals (e.g., a typical transceiver [16] is configured with less than 50 instructions), but their size could be expanded for particular scenarios.

In the case of the *static* parameters, we define four:

- reg_reset: To declare the register address that represents a reset instruction in each peripheral.
- cmd_write: This parameter is used to introduce the *write command* value for peripherals that need it as explained in Section 4.1.
- cmd_read: This is similar to the previous one, but this is the command for reading operations. If no *command* is needed in a peripheral, it has to be filled with zeros.
- i2c_add: This parameter is used to define the address of the peripherals that are attached to the MCU through I${}^{2}$C protocol.

**Figure 7:**
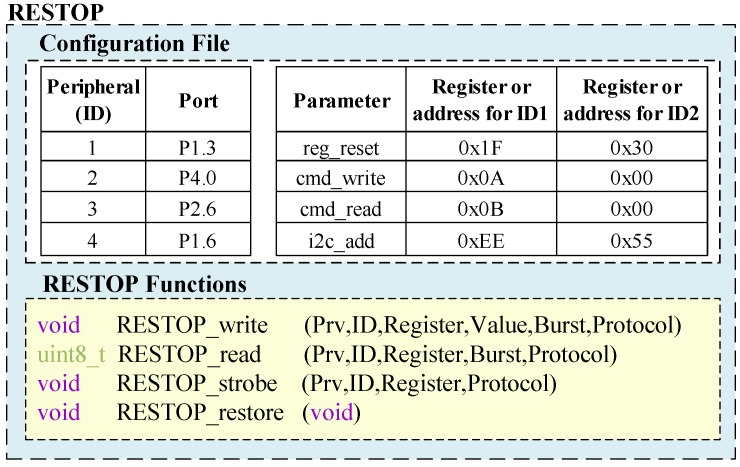
Configuration file with example values and the functions description.

The *static* parameters are declared in a configuration file unlike the *dynamic* ones, which are saved in the history table and entered by the user through the generic functions (except *R*, *Next* and *Previous*, which are defined by RESTOP). To reset a peripheral, the user not only has to use the *RESTOP_write()* function but also declare the register address in *reg_reset*. As mentioned in Section 3.1, *Reset* is a *write* instruction that when issued causes RESTOP to delete the saved instructions that correspond to the reset peripheral. *cmd_write* and *cmd_read* have to be filled with zeros for those peripherals that do not need a *command* parameter (described in Section 3.1). If an I${}^{2}$C peripheral is attached to the system, its address has to be written in *i2c_add*, if not, this parameter has to be filled with zeros as well. Figure 7 shows the *configuration file* with example values and the description of the *dynamic* parameters that each generic function requires. The *configuration file* also includes the microcontroller ports where the peripherals are attached. For example, if a user connects a peripheral to port P1.3, it will be marked with the peripheral identification ID1.

### 4.3. Instruction History Table

**Figure 8:**
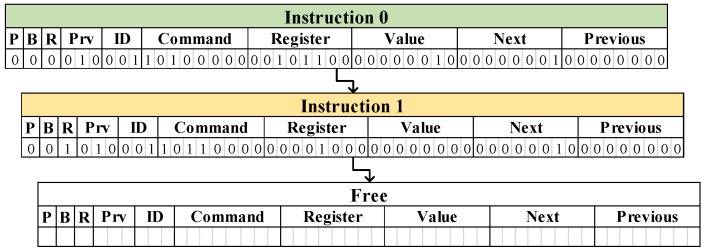
Instruction history table of two saved instructions.

As already mentioned in Section 4.2, RESTOP requires an *instruction history table* to which it can save the data exchanged between the MCU and the peripherals. It is based on a linked list in which each element corresponds to a peripheral instruction. A static array of structures can simplify the implementation of this table, as allocating memory dynamically may substantially increase time and memory overheads [18,19]. The maximum size of the table (i.e., the number of available locations where the instructions are saved) is defined by the user in the *configuration file*. Figure 8 shows an example of the history table with two saved instructions. From the values showed in the figure, the *static* parameters and the generic functions for the two saved instructions would be as follows:

- reg_reset [] = {0x1F}
- cmd_write [] = {0x0A}
- cmd_read [] = {0x0B}
- i2c_add [] = {0x00}
- RESTOP_write(2,1,0x2C,0x02,0,0)
- RESTOP_read(2,1,0x08,0,0)

In this example, the value of the *Protocol* flag (*P* = 0) indicates both instructions correspond to the same SPI peripheral, which is connected to the P1.3 port (*ID* = 1). Therefore, the parameter *i2c_add* is filled with zeros. The *Burst* flag (*B*) is zero which means these are not *burst* instructions. According with the *Read* flag (*R*), the first instruction is to *write* on the peripheral (*R* = 0) and the second is to *read* from it (*R* = 1). The *Prv* flag value is 2 in both saved instructions, which means that they would replace any similar instruction (same peripheral, command and register) previously saved, but they can also be replaced if a similar instruction is later issued (*Preserve* bit = 0). The attached peripheral needs a *command* to indicate when the instruction is to write (0x0A) and when to read (0x0B). If an instruction was issued with a register value of 0x1F, RESTOP would apply a reset in the peripheral and delete the two instructions from the table.

## 5. Experimental Validation

**Figure 9:**
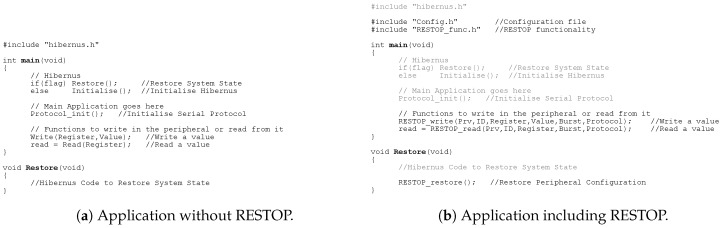
Example code of how to use RESTOP in an application, including Hibernus, showing: (**a**) code without RESTOP; and (**b**) including RESTOP.

RESTOP has been practically implemented and experimentally validated. To allow computation to span across power cycles, we combined RESTOP with Hibernus [6]. This solution was chosen because it is platform and application agnostic, and has excellent performance in terms of energy and time overhead [20]. However, we believe that RESTOP can be integrated with any other software approach for transient computing. Figure 9 shows an example application before (Figure 9a) and after (Figure 9b) incorporating the proposed middleware. The example code includes Hibernus to retain the system state between power outages. The inclusion of RESTOP in an application is simple. The developer only has to import the configuration file (*Config.h*) and the library that contains RESTOP functionality (RESTOP_func.h), and use the RESTOP functions (described in Section 4.1) to configure the peripherals and read data from them. In order to restore the peripheral state after a power outage, the RESTOP restore function has to be included in the restore routine of the transient approach as shown in Figure 9b.

The voltage threshold at which Hibernus restores the system state ($V_{R}$) has to be adjusted because now the system incorporates external peripherals whose state is restored as well. Therefore, we describe how $V_{R}$ is modified for Hibernus, which is used in our validation (a similar modification would need to be made for other approaches). In this sense, it is necessary to first calculate the amount of energy required to restore the state of attached peripherals ($E_{r\_ps}$), which is given by:

(1) $$E_{r\_ps}=\sum_{i=1}^{n}\left(P_{p_{i}}\sum_{j=1}^{m_{i}}T_{p_{i}inst_{j}}\right)$$

where *n* is the number of attached peripherals, $P_{p_{i}}$ is the power consumed by the system while undertaking serial communications with each peripheral, $m_{i}$ is the number of saved instructions for each attached peripheral and $T_{p_{i}inst_{j}}$ is the time taken by the system to issue each instruction to the peripheral. These parameters (power and time) may be obtained from datasheets, or measured experimentally. The time varies from one instruction to another depending on the data rate of each peripheral and the number of bytes that are transmitted for each instruction. In Section 4.1, we detailed how the number of parameters that are transmitted for each instruction (i.e., one parameter is equal to one byte) varies by peripheral. It is important to mention that Equation (1) only accounts for the power consumption of the MCU. The effect of issuing the instructions may cause additional energy to be consumed by the external peripherals, e.g., a restoration of state causing a wireless transceiver to make an energy-intensive radio transmission. This is not currently modeled, but is a potential area of future investigation.

Once $E_{r\_ps}$ is calculated, and considering the energy required to restore the system state ($E_{r\_sys}$ [6]), $V_{R}$ can be calculated as follows:

(2) $$V_{R}=\sqrt{\frac{2(E_{r\_sys}+E_{r\_ps})}{C}+V_{min}^{2}}$$

where $V_{min}$ is the minimum voltage required by the system to operate and *C* is the total capacitance on the supply lines, which can be used as an energy buffer. The process of calculating $E_{r\_ps}$ and adjusting $V_{R}$ is performed at the beginning of the snapshotting routine, in order to guarantee that the restore threshold is properly set before a power failure occurs. Although Equation (1) is performed once per supply interruption, a running total of $T_{p_{i}inst_{j}}$ is updated each time a peripheral instruction is saved. This reduces the complexity of the calculation that needs to be performed at the start of the snapshotting procedure. Thus, $V_{R}$ can be dynamically adjusted considering the number of saved instructions (provided by RESTOP) for each attached peripheral.

**Figure 10:**
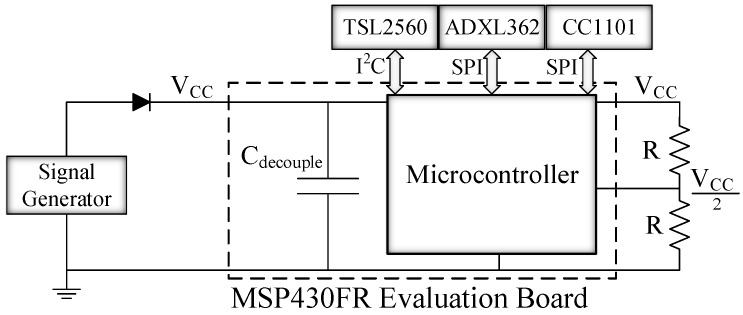
Schematic of the test platform, including the external peripherals.

An important concern is the size of *C*. Transient computing schemes commonly use only the system decoupling capacitance, $C_{decouple}$ (Figure 10), but this could be insufficient in systems interfacing with external peripherals. It may be necessary to introduce additional capacitance to deliver reliable operation. To do this, and for design purposes only, the worst case of energy used for restoring the peripheral state ($E_{r\_max}$) has to be calculated. In this sense, Equation (1) is simplified as follows:

(3) $$E_{r\_max}=P_{p\_max}\cdot n_{inst}\cdot T_{p\_max}$$

where $P_{p\_max}$ corresponds to the maximum power consumed by the system when an instruction is issued, $n_{inst}$ is the maximum number of instructions than can be saved in the *instruction history table* and $T_{p\_max}$ is the *longest* time taken to issue a single instruction. Once $E_{r\_max}$ is obtained, and with knowledge of the $V_{min}$ and $V_{max}$ (the system’s maximum operating voltage), the required *C* can be calculated as:

(4) $$C\geq \frac{2(E_{r\_sys}+E_{r\_max})}{V_{max}^{2}-V_{min}^{2}}$$

If *C* > $C_{decouple}$, RESTOP will require additional capacitance to supplement the decoupling capacitance. However, no other hardware changes are required.

Figure 10 shows the experimental set-up which consists of a test board and three peripherals. The chosen board was the MSP-EXP430FR5739 [21], which contains an MCU with FRAM, an on-chip comparator and supports SPI and I${}^{2}$C communication protocols. The comparator is used by Hibernus to monitor the input voltage. It was configured with an on-chip variable reference voltage generator and an external voltage divider (*R* = 1 MΩ) giving *V${}_{CC}$*/2 as input. We considered three different external peripherals: an ADXL362 digital accelerometer [17], a TSL2560 digital luminosity sensor [15] and a CC1101 radio transceiver [16]. The accelerometer and the transceiver are attached to the MCU through SPI, while the luminosity sensor is accessed via I${}^{2}$C. Each peripheral was tested separately (i.e., only one peripheral is used for each of the tests), giving three different scenarios in total. *C*${}_{decouple}$ represents the total decoupling capacitance of the board, which is 20 μF. To verify whether additional capacitance was needed, we evaluated the worst case energy use for each of the attached peripherals and the minimum capacitance needed for each scenario; the results are listed in Table 3.

It was therefore concluded that *C*${}_{decouple}$ was sufficient for all cases, and hence no additional capacitance was needed. The whole system was powered by two different signals:

1. A half-wave rectified sinusoidal signal with ±3.4 V amplitude operating at a frequency of 6 Hz, to emulate an intermittent source, in order to validate whether RESTOP is able to retain the peripheral’s state between power failures.
2. A square wave signal with 3.4 V amplitude and variable duty cycle, sweeping the active time from 10 ms to 100 ms, to measure the time overhead caused by RESTOP with respect to the total application execution time.

The aim of these variable signals is to emulate intermittent sources. Behaviour with a real EH source was not evaluated, as this has already been demonstrated for Hibernus-based systems in [6,7,20].

### 5.1. Accelerometer

**Figure 11:**
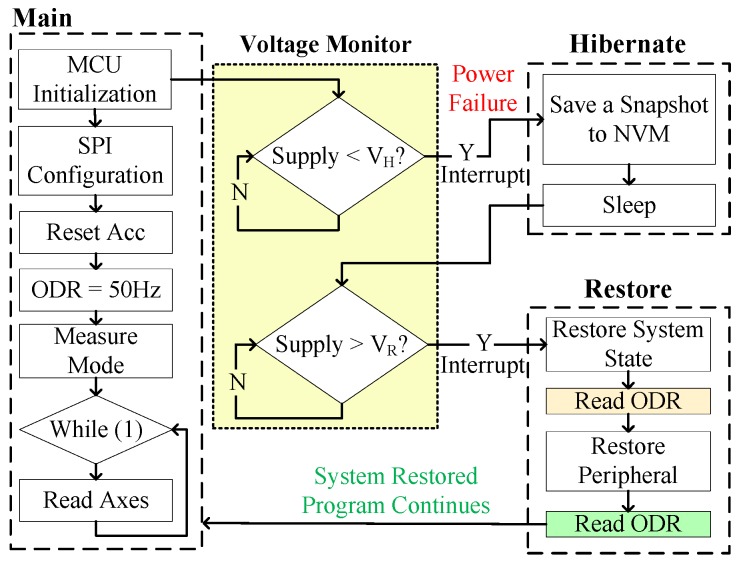
Testing routine to validate RESTOP with the accelerometer.

**Figure 12:**
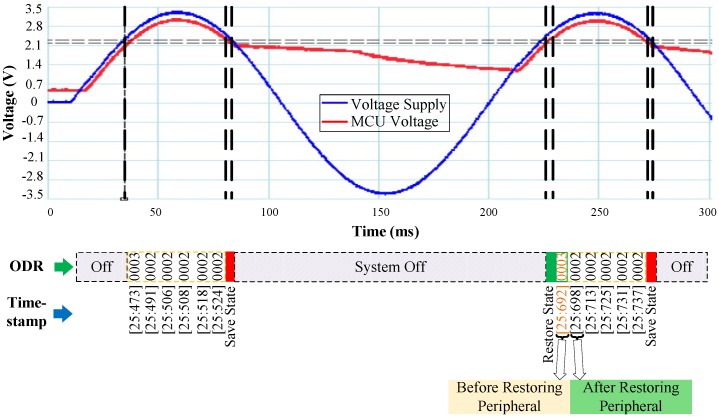
Operation of the accelerometer testing routine. After the power failure, ODR is read before and after RESTOP restores the accelerometer state.

To validate the proper operation of RESTOP with the accelerometer, we implemented an application that changes the output data rate (ODR) to reduce the current consumption of the sensor. As shown in Figure 11, after configuring the SPI protocol, the accelerometer is reset and the ODR is set to 50 Hz (ODR = 0x02). Then, the accelerometer is configured in measurement mode and the application enters in a loop where the three axes are read to detect movement at each iteration. During the time the program is running inside the loop, a voltage drop occurs and the snapshotting routing of Hibernus is called. There, $E_{r\_ps}$ is calculated using Equation (1) and the obtained value is 0.6 μJ. Substituting it in Equation (2) and considering $E_{r\_sys}$ = 5.7 μJ [6], the new restore threshold is set to 2.15 V. After $V_{R}$ is adjusted, the system state is saved in NVM. Later, when the power is restored, an ODR reading is taken before and after restoring the accelerometer state. This step was purely for testing purposes in order to check RESTOP operation: the ODR reads would not be needed in a real application. Thus, the ODR value read before restoring has to be the default (ODR = 0x03), whilst the value after restoring has to be the same as before the power failure (ODR = 0x02). As we can see in Figure 12, the value read before restoring the peripheral state is the default (ODR = 0x03), but once it is restored, the ODR value is the same as before the interrupt. This shows that RESTOP is able to restore the accelerometer state.

### 5.2. Luminosity Sensor

**Figure 13:**
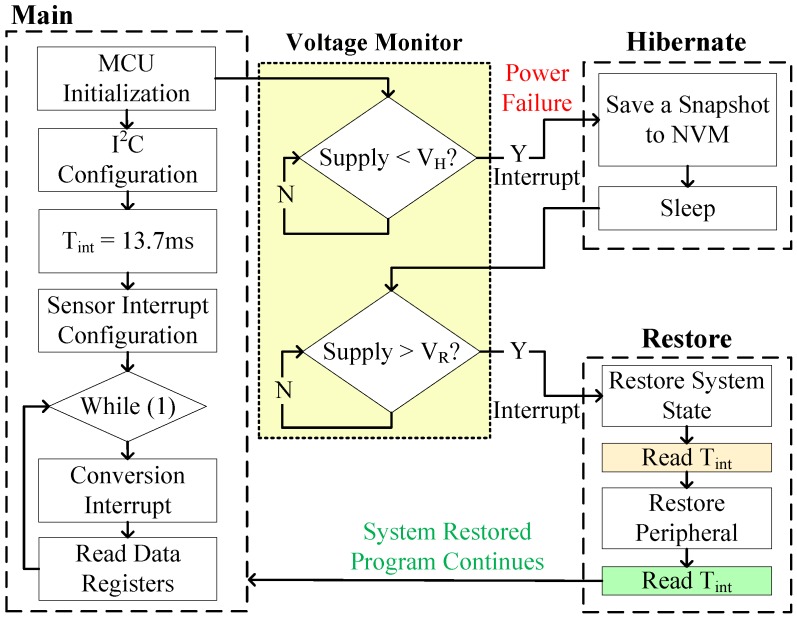
Testing routine to validate RESTOP with the luminosity sensor.

**Figure 14:**
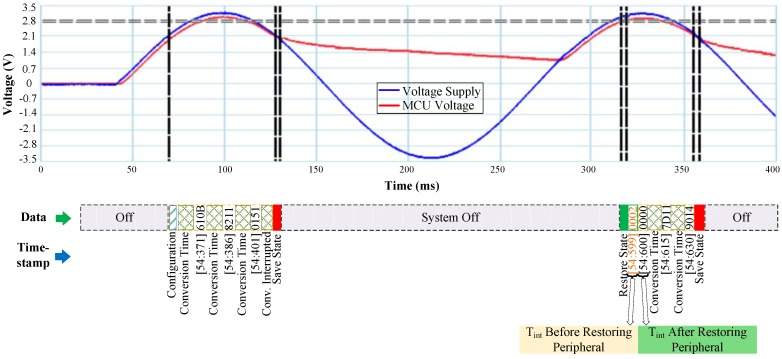
Operation of the luminosity sensor in an intermittently-powered system. After the power failure, the timing registers are configured as before the interruption.

RESTOP was tested with a luminosity sensor to validate the proposed middleware with an I${}^{2}$C peripheral. Figure 13 shows the test algorithm, which consists of initializing the MCU, configuring the I${}^{2}$C protocol, and then changing the integration time ($T_{int}$) from the default value (400 ms) to 13.7 ms. $T_{int}$ defines the time after which the ADC channels begin a conversion. Once the integration time was changed, an end-of-conversion signal is configured in order to generate an interrupt when an ADC conversion is completed. Thus, the light intensity value is available in the data registers after 13.7 ms. Figure 14 shows the experimental results. After configuring the sensor, the light intensity is continuously read until the input voltage drops and the snapshotting routine is executed. In the same way as with the accelerometer, $E_{r\_ps}$ and $V_{R}$ are calculated. However, for the luminosity sensor, the minimum operating voltage is 2.6 V, which is then defined as $V_{min}$ in Equation (2) (unlike the accelerometer’s, which is 2 V); therefore, the obtained values for $E_{r\_ps}$ and $V_{R}$ are 1.72 μJ and 2.74 V, respectively. To validate the proper operation of RESTOP, the integration time register is read before and after restoring the peripheral state. As we can see in Figure 14, the value read before restoring the peripheral configuration is $T_{int}$ = 0x02 corresponding to an integration time of 400 ms. Then, when the peripheral state is restored, the value read is $T_{int}$ = 0x00, which corresponds to 13.7 ms. This can also be proved because the end-of-conversion interrupt signal of the sensor is enabled every 13.7 ms, which means the sensor’s configuration was successfully restored by RESTOP.

### 5.3. Transceiver

**Figure 15:**
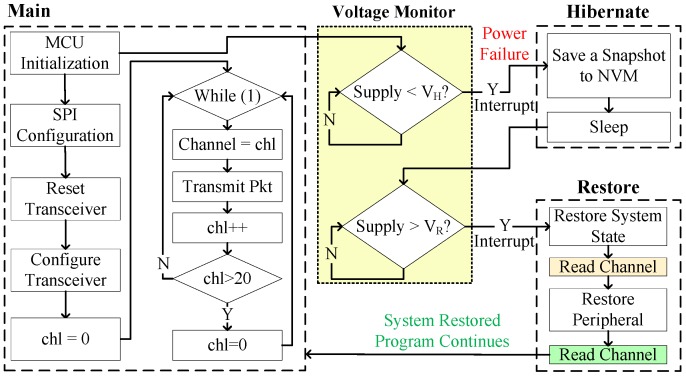
Testing routine to validate RESTOP with the transceiver.

**Figure 16:**
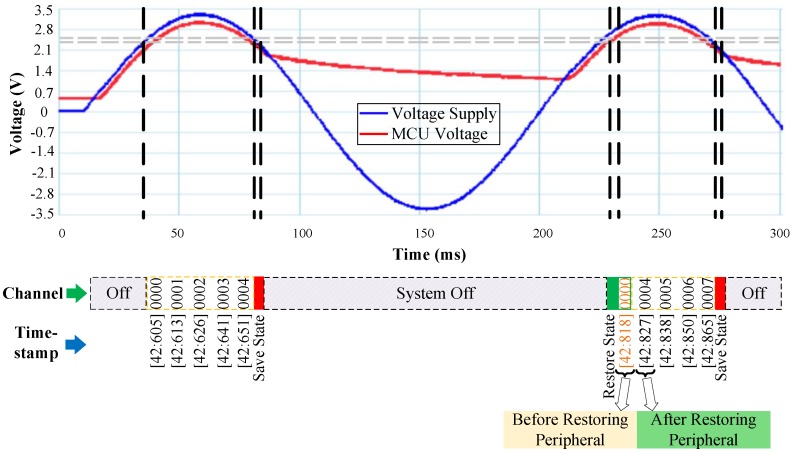
Operation of the transceiver testing routine. After the power failure, the transmission channel number is read before and after RESTOP restores the transceiver state.

The operation of RESTOP was also validated with a CC1101 radio transceiver. Exclusively for debugging purposes, we implemented a routine (Figure 15) that initializes the MCU, configures the SPI protocol, resets and configures the peripheral and then, inside an infinite loop, the program changes the transmission channel (from 0 to 20) and sends a packet at each cycle. The idea is that we can check the channel number before the power failure, and before and after restoring the peripheral state. This is to verify whether the transceiver configuration is restored after a power outage and in consequence the channel number is retained. Figure 16 shows the experimental results of RESTOP with the transceiver. When the input voltage drops, the channel number read is 4. Then, inside the snapshotting routine, the energy required to restore the transceiver state is calculated. The obtained value is 8.43 μJ, which is used to calculate $V_{R}$ = 2.33 V. When the supply voltage rises above $V_{R}$, and before the peripheral state is restored, the channel number read is 0, which is the default value. Then, RESTOP restores the peripheral state and the channel number read is 4, which is the same channel as before the power failure.

### 5.4. Time Overhead

To analyse the time overhead caused by RESTOP in an intermittently-powered system, we implemented three applications that run under two different scenarios powered by a square wave signal with 3.4 V amplitude and variable duty cycle (from 10 ms to 100 ms). In the first scenario, the peripherals are accessed without using RESTOP (restarting the peripheral’s state from scratch after each power failure), while, in the second scenario, our middleware is included. Each application consists of reading data sampled by the luminosity sensor, and reading data from the accelerometer (ACC) and processing it with a Fast Fourier Transform (FFT). Then, the sampled and processed data is transmitted through the radio transceiver. The difference in the applications is the number of samples (32, 64 and 128) that are obtained from the accelerometer and processed by the FFT.

Table 4 shows the time needed to access the peripherals with and without RESTOP. The proposed middleware saves and executes all the *write* instructions to configure the three peripherals and the *read* instructions to get the data from the sensors. The time taken by the FFT is the same in both scenarios because RESTOP is transparent for this task. In the case of the luminosity sensor and the transceiver, they spend the same time in all the applications because they operate only once per case, unlike the accelerometer which takes different amounts of samples. Table 4 also presents the total time spent to complete the FFT, including the time to snapshot and restore both the system state and the peripheral configuration. The last column (at the right side) indicates the time overhead caused by RESTOP on the whole application with different active times. RESTOP causes a time overhead of about 15% when configuring a peripheral. However, this represents a maximum overhead of 0.82% during complete execution of our typical sensing application and is substantially lower than the existing approach *Sytare* [9], which causes a time overhead of up to 137% (30 μs per peripheral instruction) when configuring a radio transceiver. Moreover, the time overhead caused by RESTOP will decrease further as the ratio of the peripheral instructions: normal operation decreases.

## 6. Conclusions and Future Work

We have proposed RESTOP, a new approach to retain the state of peripherals that communicate with an MCU through a digital interface, in transient computing systems. The presented middleware provides generic functions to read data from the external peripherals or write to them, and keeps track of and saves the transmitted configuration data into the instruction history table from where the peripheral state is restored after a power failure. With these characteristics, RESTOP can be integrated into any of the existing approaches for transient computing and, unlike existing approaches, it is able to operate generically with multiple devices that communicate with the MCU through different protocols such as SPI or I${}^{2}$C. RESTOP has been validated with a digital accelerometer (SPI), a luminosity sensor (I${}^{2}$C) and a radio transceiver (SPI) in an intermittently-powered system. Results demonstrate that RESTOP is capable of restoring the peripheral state after power outages causing a time overhead to the application of up to 0.82% during complete execution of our typical sensing application, which is considerably lower than that caused by existing approaches.

**Figure 17:**
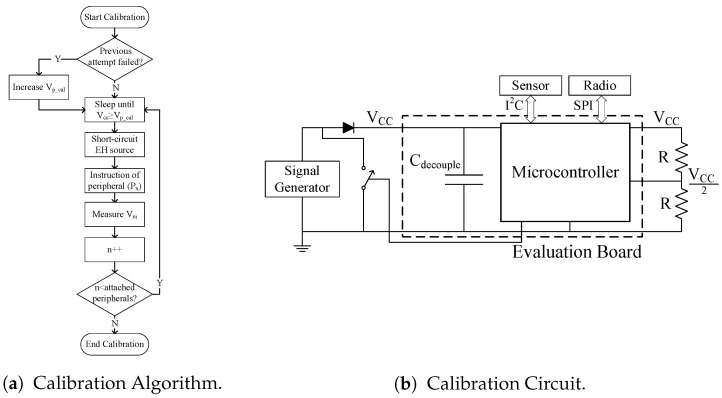
Calibration routine to measure the energy consumed by external peripherals when executing an instruction, showing: (**a**) the algorithm; and (**b**) the circuit schematic.

In this work, the energy cost of restoring the state of peripherals was modeled, but any additional energy used by the peripherals (e.g., a restored instruction that then triggers a radio transmission) was not taken into account. Hence, in the future we are looking to account for the energy requirements of the complete system, i.e., not just the MCU. A potential solution to this is to implement a calibration routine similar to that used in Hibernus++ [7], but in this case for measuring the energy consumed when executing each peripheral instruction. As shown in Figure 17a, the calibration routine would wait for the supply voltage to reach the calibration voltage ($V_{p\_cal}$). When this voltage is reached, the EH source would be short-circuited by closing the switch in Figure 17b, and an instruction is issued to the peripheral. The drop in supply voltage caused by issuing and executing the instruction is given by $V_{p\_cal}$ - $V_{m}$, where $V_{m}$ is the voltage measured after the instruction has been completed. This process would be executed once per each attached peripheral.

Another package of work is to integrate RESTOP with a generic operating system, e.g., the ARM mbed OS which has already been demonstrated with Hibernus [22]. Operating systems have components and abstractions for peripheral interface, which could be combined with RESTOP. This would further increase the accessibility of transient computing systems and promote standardization.

## Figures and Tables

**Table 1:** Dynamic parameters to be considered for describing a peripheral instruction.

Parameter	Size (Bits)	Definition
Protocol	1	Serial Protocol of Peripheral
Burst	1	Burst instruction
Read	1	Read or write instruction
Prv	3	Preserve flag
ID	3	Peripheral identification
Register	8	Address to be accessed
Value	8	Value to be written in the register
Next	8	Pointer to the next instruction
Previous	8	Pointer to the previous instruction

**Table 2:** Values that Prv flag can have.

Bit 2	Bit 1	Bit 0	Criteria
0	0	0	Not-save
0	0	1	Save
0	1	0	Save-but-replace
1	0	1	Save and Preserve
1	1	0	Save-but-replace and Preserve

**Table 3:** Worst case energy use for each peripheral, and the minimum capacitance needed.

Peripheral	E${}_{r\_max}$ (μJ)	*C* (μF)
Accelerometer	1.40	1.58
Luminosity	2.87	2.76
Transceiver	9.37	3.36

**Table 4:** Time overhead caused by RESTOP in a system with three external peripherals.

Active time (ms)	No. Sampl.	No. Exec. Inst’s	No. S’shot	No. Rest.	Time (ms)									O’head (%)
						Without RESTOP				With RESTOP				
					FFT	Lum.	Acc.	Xcvr.	Total	Lum.	Acc.	Xcvr.	Total	
10	32	80	2	2	19.7	-	0.72	1.05	26.96	-	0.78	1.21	27.18	0.82
10	64	112	6	6	47.3	-	1.43	1.05	66.19	-	1.49	1.21	66.41	0.33
10	128	176	14	14	100	-	2.97	1.05	142.4	-	3.03	1.21	142.6	0.15
40	32	85	0	0	19.7	14.1	0.72	1.05	35.58	14.2	0.78	1.21	35.86	0.79
40	64	117	1	1	47.3	14.1	1.43	1.05	66.59	14.2	1.49	1.21	66.87	0.42
40	128	181	3	3	100	14.1	2.97	1.05	126.3	14.2	3.03	1.21	126.6	0.22
70	32	85	0	0	19.7	14.1	0.72	1.05	35.58	14.2	0.78	1.21	35.86	0.79
70	64	117	0	0	47.3	14.1	1.43	1.05	63.85	14.2	1.49	1.21	64.13	0.44
70	128	181	1	1	100	14.1	2.97	1.05	120.9	14.2	3.03	1.21	121.1	0.23
100	32	85	0	0	19.7	14.1	0.72	1.05	35.58	14.2	0.78	1.21	35.86	0.79
100	64	117	0	0	47.3	14.1	1.43	1.05	63.85	14.2	1.49	1.21	64.13	0.44
100	128	181	1	1	100	14.1	2.97	1.05	120.9	14.2	3.03	1.21	121.1	0.23
